# Advances in Systemic Lupus Erythematosus (SLE): A case for optimism

**DOI:** 10.31138/mjr.28.1.1

**Published:** 2017-03-28

**Authors:** Antonis Fanouriakis, Dimitrios T. Boumpas

**Affiliations:** 1 Joint Rheumatology Program, Medical School, National and Kapodestrian University of Athens and 4^th^ Department of Medicine, Attikon University Hospital, Athens, Greece

SLE is a heterogeneous, multi-organ autoimmune disease with a wide range of clinical and laboratory abnormalities. The hallmark of lupus is the generation of autoantibodies directed against a number of nuclear constituents, including double-stranded DNA (dsDNA), chromatin, small nuclear ribonucleoproteins, and antibodies against negatively charged phospholipids. Disease prevalence typically ranges from 0.05–0.15% with a strong female predominance (7 to 12:1).^1,2^ The etiology remains incompletely understood, although several components of the innate and adaptive immunity and various factors (hormonal, environmental, genetic) contribute to the disease. Numerous studies have revealed the involvement of several cell types in lupus, including lymphocytes, monocytes, plasmacytoid dendritic cells, neutrophils and endothelial cells. The multiorgan involvement and the lack of specificity in its manifestations can make the diagnosis of SLE at early stages challenging.^3^

SLE is characterized by alternating phases of quiescence and exacerbations, the latter associated with an imminent risk for accrual of irreversible organ damage. To date, there are no accurate biomarkers for assessment of disease activity or for the prediction of disease severity, response to treatment or flares. Existing treatments include broad-spectrum cytotoxic and immunosuppressive agents that lack specificity, are associated with significant adverse events and achieve long-term disease remission in less than 20–30% of patients (with nearly one third of them relapsing following initial remission).

Involvement of major organs with extensive microvasculature, such as the kidneys and the central nervous system (CNS), and comorbidities, such as cardiovascular disease and infections, are major drivers of morbidity, mortality and cost of disease in SLE.

In this issue of the *Mediterranean Journal of Rheumatology,* three excellent review papers critically examine major aspects of SLE, namely: a) inhibition of interferon-α (IFN-α) and its promise in lupus therapeutics; b) management of SLE thrombocytopenia; and c) lupus flares - risk factors, diagnosis and preventive strategies.^4–6^

## IFN-α IN SLE

Historically, the involvement of the complement system - an integral part of the innate immune response - in the pathogenesis of lupus was recognized early. However, emphasis quickly shifted towards adaptive immunity, with scientists concentrating on the adaptive immune response (autoantigens, autoreactive T cells and auto-antibodies). Similarly, the detection of interferon alpha (IFN-α), another key mediator of innate immunity, in the sera of active lupus patients by Hooks and Moutsopoulos in 1979, was poorly understood and rather ignored for many years. In recent years, the realization that a) endogenous ligands (“stressors”) derived from a “stressed” host can be potent inducers of inflammatory mediators, and b) an intimate cross-talk exists between the innate and the specific immune response, has motivated investigators to take a closer look at innate immunity, especially IFN-α, as both a key to better understanding of lupus pathogenesis, a biomarker of disease activity and a therapeutic target.^7^

Type I IFNs are secreted by plasmacytoid dendritic cells (pDCs) and drive almost every aspect of SLE pathogenesis, including monocyte activation, lymphocyte differentiation and activation and endothelial injury.^8^ Inherited mutations causing activation of the type I IFN pathway result in a phenotype of systemic autoimmunity, which encompasses some of the manifestations of lupus. Patients with lupus have increased expression of IFN-stimulated genes in the peripheral blood mononuclear cells, which correlates with severe disease, especially renal involvement. Recent therapeutic approaches targeting the IFN system in SLE include monoclonal antibodies directly targeting IFN-α (sifalimumab, rontalizumab) or its receptor (MEDI-546, AIA22, and anifrolumab), or the use of interferon alpha kinoid to stimulate endogenous production of anti-IFN antibodies in lupus. Other drugs used in lupus, such as hydroxychloroquine and bortezomib, reduce circulating levels of type I IFNs. Newer therapeutic strategies investigated in preclinical models of lupus, which reduce the production of type I IFNs, include dihydroartemisinin, Bruton’s tyrosine kinase antagonists, Bcl-2 antagonists and sphingosine-1 phosphate agonists.^4^ Together, the results from these trials have confirmed the rationale of targeting IFN-a in SLE, with significant reductions in lupus activity, but also an increased risk for herpes zoster infections. Our prediction is that targeting IFN-α may prove useful as an add-on therapy to standard regimens in SLE, to accomplish deeper remission and prevent flares.

## LUPUS THROMBOCYTOPENIA

Severe, refractory lupus thrombocytopenia, albeit rare, is a major challenge in SLE and patients with severe thrombocytopenia have a higher mortality. With the exception of immune thrombocytopenia (ITP) in children, a significant proportion of adults with ITP, especially those who are ANA positive, may have unrecognized SLE or develop lupus later. These ITP patients with autoimmune features (AIF-ITP) tend to be positive for ANA and have lower complement levels.^9^ For the most part, acute therapy of severe thrombocytopenia or for the bleeding patient is the same as in ITP and includes high-dose glucocorticoids and IVIG, but response is often short-lived and requires maintenance with other immunosuppressive drugs (hydroxychloroquine, danazol, azathioprine, cyclosporine, mycophenolate mofetil, cyclophosphamide), which may also control the disease in other major organs, such as kidneys and central nervous system. Although splenectomy is an option for refractory patients, in SLE this usually follows immunosuppressive therapy. New agents, such as B-cell depleting monoclonal antibodies and thrombopoietin receptor agonists, are emerging as steroid-sparing modalities, but their efficacy/safety ratio requires further documentation. In reference to B-cell depleting antibodies, such as rituximab or obinutuzumab, available data suggest an efficacy > 60% in lupus thrombocytopenia, but responses are often temporary. Newer, type II anti-CD20 mAbs like obinutuzumab, may prove more effective in this context, due to a more robust B-cell depletion. Romiplostim and eltrombopag are thrombopoietin receptor agonists, which mediate the proliferation and differentiation of megakaryocyte progenitors, maturation of megakaryocytes and increased platelet production; currently, they are used as second-line agents in ITP. However, in lupus patients with thrombophilia (e.g., those with circulating antiphospholipid antibodies), concerns have been raised regarding their potential increased thrombophilic risk.^5^

## FLARES

Initial studies on lupus therapy concentrated on the control of disease activity and induction of remission. However, it soon became clear that lupus patients often experience disease exacerbations (flares) of varying severity; thus, prevention of flares emerged as a distinct therapeutic target in SLE, in trials involving cyclophosphamide, azathioprine, mycophenolate mofetil and belimumab.^1^ Flares carry a risk for irreversible damage and expose patients to side effects of the intensified immunosuppressive therapy. Indeed, both the number and the severity of SLE flares have been correlated with damage accrual.^10^

Despite intense efforts to identify reliable *biomarkers* for the prediction and early recognition of flare (including the widely-used serum complement fragments and anti-dsDNA autoantibodies), no such markers exist, and diagnosis of flares is primarily based on clinical grounds. As the authors of this review point out,^6^ serologic activity *per se* (i.e., clinically quiescent, serologically active SLE) does not provide sufficient grounds for intensifying therapy but calls for a closer monitoring. Well-documented strategies to predict flare in SLE include early treatment aiming at the achievement of deep, sustained remission, maintaining immunosuppressive or biologic therapy for at least 5 years (or 2–3 years beyond remission, if possible with complete withdrawing of glucocorticoids), and the use of hydroxychloroquine.^6^ The importance of compliance with SLE therapy has received increased attention in recent years, with non-compliance rates especially for hydroxychloroquine, estimated to be as high as 30%.^11^

## CONCLUSION

Despite considerable progress, much remains to be learned about SLE and its management. Recent advances in high-throughput technologies have offered an initial glimpse in understanding what predisposes to SLE, as well as the genes that determine particular organ involvement and severity, with the prospect of identifying reliable biomarkers for diagnosis, monitoring and personalized therapy.^12,13^ Since off-treatment prolonged remission in lupus is rare, new definitions of low-disease activity may provide a more realistic target for therapy, in order to decrease the risk of flares and accrual of damage.^14^ Combination, multitargeted therapies for lupus nephritis combining glucocorticoids, tacrolimus and mycophenolate mofetil may expedite remission in patients with severe disease; however, these data need to be confirmed in non-Asian populations.^15^

Prevention of damage accrual is now considered an important frontier in the management of SLE, with current efforts focusing on minimizing the use of glucocorticoids (as they represent major determinants of damage in lupus) and decreasing rate and severity of flares, by means of new conventional and biologic therapies. Among all aspects of SLE, neuropsychiatric disease - probably the least understood aspect of SLE - has also emerged as a major driver of SLE damage, with several groups attempting to capitalize on the benefits of novel imaging techniques, in order to allow for more targeted therapy. These advances, together with the data presented in the current issue of the *Mediterranean Journal of Rheumatology*, justify a cautious optimism regarding the management of this most fascinating disease.
